# Disease burden in Serbian patients with facioscapulohumeral muscular dystrophy

**DOI:** 10.3389/fneur.2026.1720488

**Published:** 2026-03-16

**Authors:** Branislav Ralic, Noemi Albano, Vanja Viric, Andi Nuredini, Ana Arsic Azanjac, Sonja Rajic, Ana Marjanovic, Rossella Tupler, Chad Heatwole, Stojan Peric

**Affiliations:** 1 Department of Neurology, Clinical Hospital Center Zvezdara, Belgrade, Serbia; 2 Department of Biomedical, Metabolic and Neural Sciences, University of Modena and Reggio Emilia, Modena, Italy; 3 Neurology Clinic, University Clinical Center of Serbia, Belgrade, Serbia; 4 Department of Neurology, Faculty of Medical Sciences, University of Kragujevac, Kragujevac, Serbia; 5 Clinic of Neurology, University Clinical Center of Vojvodina, Vojvodina, Serbia; 6 Faculty of Medicine, University of Novi Sad, Novi Sad, Serbia; 7 Department of Neurology, University of Rochester Medical Center, Rochester, NY, United States; 8 Faculty of Medicine, University of Belgrade, Belgrade, Serbia

**Keywords:** disease burden, facioscapulohumeral muscular dystrophy, FSHD-HI, quality of life, validation

## Abstract

**Background:**

This study aimed to adapt the Facioscapulohumeral Muscular Dystrophy - Health Index (FSHD-HI) for Serbian patients with facioscapulohumeral muscular dystrophy (FSHD) in order to measure their disease burden.

**Patients and method:**

Forty-one patients with genetically confirmed FSHD1 were included in the study. Validation involved reliability analysis (internal consistency), content validity, construct validity, and criterion validity analyses. The Comprehensive Clinical Evaluation Form (CCEF) was employed to capture various FSHD phenotypes. All patients completed the Serbian version of the Short Form Health Survey (SF-36) questionnaire, serving as a generic measure of the health-related quality of life (QoL).

**Results:**

All patients found Serbian version of FSHD-HI (FSHD-HI-RS) understandable and that the language was appropriate and simple. The internal consistency of FSHD-HI-RS was excellent for the whole questionnaire (Cronbach’s alpha >0.90). Test–retest reliability met the required level (intraclass correlation coefficient 0.91). FSHD-HI scores showed significant correlations with disease duration (rho = 0.564, *p* < 0.01), muscle strength measured with Medical Research Council (MRC) sum score (rho = −0.708, *p* < 0.01), and CCEF (rho = +0.716, *p* < 0.01). FSHD-HI total score correlated significantly with the total SF-36 score (rho = −0.733, *p* < 0.01).

**Conclusion:**

Our data demonstrate that FSHD-HI-RS is an understandable, reliable, and valid measure of the disease burden in FSHD. It is easy to administer and complete, and it can capture disease-specific features that may be omitted with generic QoL questionnaires.

## Introduction

Facioscapulohumeral muscular dystrophy (FSHD) is an autosomal dominant genetic condition that causes slowly progressive muscular weakness of variable severity (1). FSHD is the second most common muscular dystrophy in adults, and overall prevalence is estimated at 1 in 20,000 inhabitants (2, 3). FSHD type 1 is genetically characterized by the shortening of the D4Z4 repeats in the subtelomeric region of the chromosome 4 (4). Studies have shown that a smaller D4Z4 repeat size is usually associated with more severe symptoms (5). The age at onset of FSHD varies from the infantile period to late in life. Variability in disease onset, progression, and severity, both between and within families, is a striking hallmark of the disease (6). Besides muscular weakness, patients may also experience a wide range of issues, such as pain, hearing loss, fatigue, and emotional distress (7).

The Facioscapulohumeral Dystrophy Health Index (FSHD-HI) is a disease-specific, patient-oriented, self-reported outcome measure designed to quantify disease burden in clinical practice and therapeutic trials (8, 9). FSHD-HI consists of relevant items and subscales identified through extensive qualitative interviews and a large cross-sectional study of FSHD patients (10). It was copyrighted in 2013 (11) and has since been used as a marker of a disease burden in many industry-sponsored and academic research studies. It has been proven as a reliable, patient-validated questionnaire with a high correlation with functional outcomes (12). It is also a valid longitudinal marker of the disease burden (9).

FSHD-HI is considered a multifactorial, highly relevant, disease-specific patient-reported outcome measure that measures the different areas of physical, mental, social, and disease-specific health most important to persons with FSHD (10). It contains 14 subscales that measure ambulation and mobility, hand function, shoulder and arm function, emotional health, back/chest/abdomen strength, fatigue, pain, eating function, ability to do activities, communication ability, satisfaction in social situations, performance in social situations, body image and cognition (13). Higher FSHD-HI score represents a higher disease burden. It is shown to be a valid mechanism to quantify multifactorial disease burden in FHSD. FSHD-HI has its short form (FSHD-HI-SF) that can be completed in less than 1 min. When time to completion is an issue, the short form can be considered as an alternative mechanism to the FSHD-HI to record and quantify a disease severity (12). FSHD-HI-SF score can also be calculated if the whole questionnaire is completed as all of the questions in the short form are also included in the full version. The validation and translation of this outcome measure has been done in many languages, including English (Australia), Dutch (Netherlands), French (France), French (Canada), German (Germany), Hindi (India), Italian (Italy), Japanese (Japan), Spanish (Spain), Spanish (US), Spanish (Latin America) (14).

In this study, we aimed to translate the FSHD-HI into Serbian, to make cultural adaptations, and assess its psychometric properties in Serbian patients with FSHD. Furthermore, disease burden in FSHD patients was analyzed using this valid marker.

## Patients and method

The study was approved by the Ethical Committee of the University Clinical Center of Serbia, and all patients gave their informed consent before entering the study. Participants in this study were patients with genetically confirmed FSHD1 recruited from the database of the University Clinical Center of Serbia in Belgrade. For all patients D4Z4 segment length was available. All of them were older than 18. From March 2023 until March 2024, we attempted to reach all 85 patients from the database to take part in this study. Overall, 44 patients responded to our request, and they were clinically examined. The remainder of the participants were either lost to follow-up (16 patients), not willing to participate in the study (12 patients), had another concomitant severe disease that prevented them from participating with the testing (4 patients), or were later confirmed to have another significant disease that may impact the assessments (9 patients). Three patients did not complete the whole questionnaire and they were excluded from the analysis. In total, 41 patients were analyzed.

For the purpose of this study, we obtained permission from the University of Rochester which owns FSHD-HI copyrights for the Serbian translation of the questionnaire. The authors of this paper have received direct permission to include descriptions and statements regarding the content and format of the copyrighted FSHD-HI. The University of Rochester is the exclusive owner of the FSHD-HI including the translated Serbian version (FSHD-HI-RS) and rights therein.

**Figure 1:**
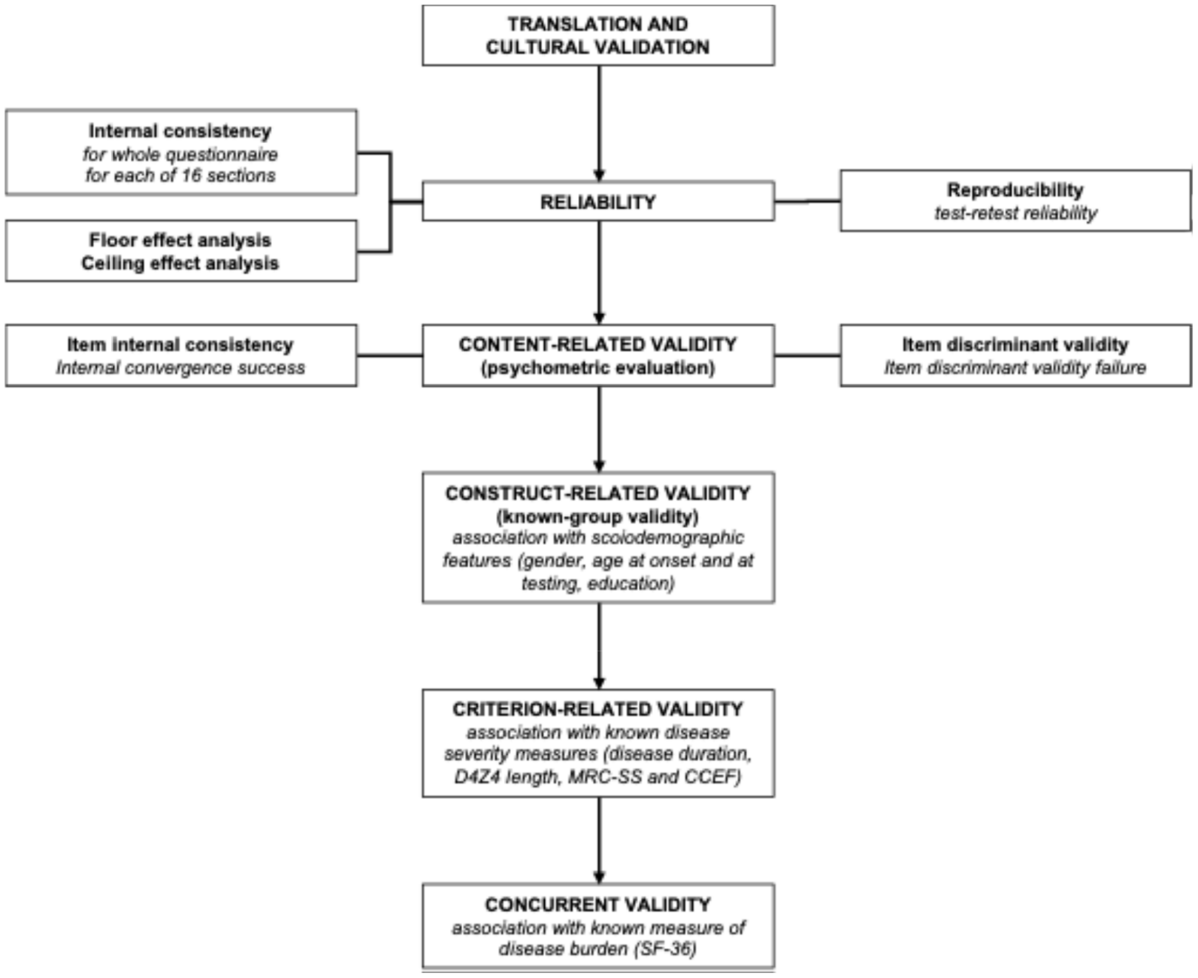
Flowchart representing steps used to validate FSHD-HI questionnaire. MRC-SS, Medical Research Council sum score; CCEF, Comprehensive Clinical Evaluation Form; SF-36, Serbian version of the Short Form (36) Health Survey.

Detailed steps conducted in the process of validation are given in Figure 1 and described below in the text. A standardized translation process was used to translate the original version of the FSHD-HI into Serbian. The translation process itself considered that two native Serbian speakers, experts in neuromuscular disorders (authors of this manuscript: B. R. and S. P.), independently translated the English version of FSHD-HI. Then the translations were compared and all the differences were resolved using a consensus between the investigators. The reconciled Serbian version was back translated into English by two professional translators who were blinded to the original version. Following thorough discussions within the translation team, a draft version of the FSHD-HI-RS Serbian was created and tested among Serbian patients to ensure that the patients appropriately understood the original meaning of the items and that the items in the FSHD-HI were culturally appropriate. After translation into Serbian, a preliminary version of the questionnaire was evaluated in seven patients in order to assess their comprehension of the items. Specifically, we read each question together with the patients, discussed any uncertain items, and examined the range of possible choices for acceptability. After the patient-assisted assessment of comprehension, we reformulated and modified some of the questions in the following FSHD-HI subscales: shoulder and arm function, mobility and ambulation, activity and limitation, body image, and emotional health in order to be more clear and understandable for patients. After the modification, the final translated version of FSHD-HI-RS was created and administered to the rest of the study group.

Test–retest reliability of FSHD-HI-RS was assessed by administering it twice 10 days apart in the first 10 subjects recruited. A 10-day interval was considered short enough to minimize actual changes in FSHD condition, but long enough to reduce recall. It is also of note that these patients were not the same as those seven that took part in the primary translation and adaptation process.

Sociodemographic and clinical data were obtained for each participant. Manual muscle testing (MMT) was used to assess muscle strength by using the standard Medical Research Council (MRC) scale which scores each muscle on a scale of 0 (no muscle movement at all) to 5 (normal muscle contraction against full resistance) (15). MRC sum score (MRC-SS) included following muscle groups in the upper extremities (external rotator of upper limb, elbow flexors and extensors, common finger extensors, wrist extensors, long finger flexors, and wrist flexors) and lower extremities (hip abductors and flexors, knee flexors and extensors, plantar flexors and extensors) bilaterally, as well as neck extensors. Overall maximum score was 135 points. The Comprehensive Clinical Evaluation Form (CCEF) is used for capturing various FSHD phenotypes, from classic FSHD to individuals with incomplete phenotypes or asymptomatic carriers, as well as subjects with atypical signs for which alternative diagnoses may be suggested (16). Category A is typical FSHD phenotype, category B is incomplete phenotype (lacking either facial or scapular involvement), category C patients are asymptomatic while complex phenotypes are represented by category D. For the purpose of this study we also used CCEF score as a surrogate measure of the disease severity. In CCEF score, severity is on a 15-points scale, where 0 is asymptomatic and 15 severely affected.

All patients completed the Serbian version of the Short Form (36) Health Survey (SF-36) questionnaire as a generic measure of the health-related quality if life (17). This instrument measures eight health concepts: physical functioning (PF), role physical (RP), bodily pain (BP), general health (GH), vitality (VT), social functioning (SF), role emotional (RE) and mental health (MH). Two main scores summarize these scales: physical composite score (PCS) and mental composite score (MCS), as well as total SF-36 score. All these scores fall within a 0–100 scale, with higher scores reflecting better QoL.

### Statistical analysis

Normality of data was tested by the Kolmogorov–Smirnov test. Results were presented as mean (standard deviation) and/or median (interquartile range).

Statistical analysis for each step of validation process (Figure 1) is described below. Different Internal consistency was analyzed for the questionnaire as a whole and also for each of 16 sections using Cronbach’s alpha (the minimal standard was 0.70 for group comparisons and 0.90 for individual comparisons). Percentage of patients obtaining the top score (ceiling effect) and bottom score (floor effect) on total FSHD-HI and FSHD-HI-SF were calculated. Test–retest reliability was assessed by intraclass correlation coefficient (ICC). Minimal standard of ICC was 0.70 for group comparisons (which means that instrument gives the same results when nothing has changed in the whole cohort of patients), and 0.90 for individual comparisons (which means that instrument gives the same result when nothing has changed in an individual patient, i.e., measurement error is very low).

Item internal consistency was analyzed as Spearman’s correlation coefficient between an item in a domain and the domain score computed from all other items in the same domain (rho≥0.40 meaning appropriate inclusion of items within a specific domain). Internal convergence failure was expressed as a percentage of items with the internal correlation coefficients below 0.40. Item discriminant validity was assessed as Spearman’s correlation coefficient between an item of a section and other similar sections. Item discriminant validity failure was expressed as a percentage of items with correlations with their own scales lower than correlations with other similar scales.

Construct-related validity was assessed as an expected impact of gender, age at testing, and education on questionnaire scores. To compare the two patient groups (male vs. female), Student’s t-test was used. Spearman’s coefficient was used to assess correlations of FSHD-HI with age and education. The relationship of FSHD-HI with disease duration, D4Z4 length, MRC-SS, and CCEF score was analyzed by Spearman’s coefficient representing criterion-related validity. The association between FSHD-HI scales and total score with SF-36 domains, composite scores and the total score was analyzed by Spearman’s correlation coefficient representing concurrent validity.

In all analyses, significance testing was two-sided, with alpha set at 0.05 for statistical significance and 0.01 for high statistical significance.

## Results

Sociodemographic and clinical characteristics of the patients are listed in Table 1. All patients included in our study found FSHD-HI-RS understandable and that the language was appropriate and simple. Results on FSHD-HI are shown in Table 2.

**Table 1:** Sociodemographic and clinical characteristics of investigated FSHD patients.

Patient characteristics	All patients (*n* = 41)
Gender (%)	
Male	24 (58.0%)
Female	17 (42.0%)
Age (mean ± SD, years)	41.9 ± 16.9
Educational level (%)	
Elemenatary school	9 (22.0%)
High school	21 (51.2%)
University degree	11 (26.8%)
Employment status (%)	
Unemployed	11 (26.8%)
Employed	24 (58.5%)
Retired	6 (14.6%)
Age at onset	
(Mean ± SD, years)	20.8 ± 10.5
(Median, IQR)	18 [15–27]
Disease duration	
(Mean ± SD, years)	15.3 ± 13.6
(Median, IQR)	11 [3.5–27.5]
MRC-SS (mean ± SD)	122.5 ± 13.2
CCEF score	
(Mean ± SD, years)	5.6 ± 4.2
(Median, IQR)	5 [2–9]
D4Z4 size (mean ± SD, kilobase)	25.0 ± 7.3

MRC-SS, Medical Research Council sum score; CCEF, Comprehensive Clinical Evaluation Form.

**Table 2:** FSHD-HI scores in Serbian patients with facioscapulohumeral muscular dystrophy.

FSHD-HI score	Mean ± SD	Median [IQR]
Shoulder and arm function	38.0 ± 35.7	31.4 [3.4–68.1]
Mobility and ambulation	34.3 ± 38.4	12.3 [0–72.25]
Fatigue	34.7 ± 34.1	26.4 [0–67.9]
Cognitive function	7.9 ± 18.1	0 [0–10.5]
Activity limitation	30.0 ± 35.5	12 [1.1–62.9]
Core strength and function	29.8 ± 33.8	12.6 [1.5–56.15]
Eating function	6.2 ± 15.1	0 [0–0]
Social performance	26.9 ± 29.9	17.8 [0–53.2]
Body image	29.1 ± 30.3	22.5 [0–66.55]
Hand and finger function	15.1 ± 25.6	0 [0–25.55]
Social satisfaction	21.0 ± 26.8	4.4 [0–43.95]
Pain	22.5 ± 29.1	6.3 [0–42.6]
Emotional health	16.8 ± 21.4	4.4 [0–28.75]
Communication	10.4 ± 16.2	0 [0–16.45]
FSHD-HI total score	27.2 ± 26.6	17.9 [2.15–51.95]
FSHD-HI short form total score	28.4 ± 26.3	24.3 [2.4–49.2]

SD, standard deviation; IQR, interquantile range.

The internal consistency of FSHD-HI-RS was “excellent” for all subgroups of the question (scores above 0.90), except for subgroups Body Image and Communication with coefficients 0.612 and 0.659, respectively (“questionable” reliability), and Eating Function 0.76 (“acceptable” reliability) (Table 3). The floor effect was 9.8% for the total score and 17.1% for the Short form. The ceiling effect for both scores was absent. The intraclass correlation coefficient was 0.91 satisfying a test–retest reliability.

**Table 3:** Reliability and content-related validity of FSHD-HI (*n* = 41).

FSHD-HI domain	Internal consistency	Content-related validity		
	Cronbach’s alpha	Item internal consistency (Spearman’s rho)	Internal convergence failure (%)	Item discriminant validity failure (%)
Shoulder and arm function	0.971	0.802–0.914	0	0
Mobility and ambulation	0.979	0.776–0.967	0	13.3
Fatigue	0.955	0.671–0.953	0	14.3
Cognitive function	0.945	0.769–0.854	0	0
Activity limitation	0.988	0.771–0.921	0	41.2
Core strength and function	0.972	0.678–0.883	0	88.9
Eating function	0.764	0.618–0.858	0	0
Social performance	0.928	0.709–0.937	0	43.0
Body image	0.659	0.677–0.820	0	50.0
Hand and finger function	0.936	0.815–0.944	0	0
Social satisfaction	0.837	0.709–0.867	0	0
Pain	0.961	0.573–0.838	0	18.2
Emotional health	0.927	0.318–0.804	11.1	22.2
Communication	0.612	0.318–0.700	25.0	25.0
Total FSHD-HI score	0.993	/	/	/
Short form	0.930	/	/	/

Item internal consistency was satisfactory for all groups of questions. Internal convergence failure was 0%, except for Emotional Health and Communication scales. Item discriminant validity failure was high for Core Strength and Function, Body Image, Social Performance, and Activity Limitation.

Construct-related validity of FSHD-HI-RS is shown in Supplementary Table 1. There were no significant associations of FSHD-HI scores with gender, age, and education. Regarding criterion-related validity, FSHD-HI scores showed significant correlations with disease duration, MRC-SS, and CCEF score (Table 4). Correlation with D4Z4 length was not observed (Supplementary Table 2).

**Table 4:** Construct-related validity of FSHD-HI.

FSHD-HI domain	Disease duration	MRC-SS	CCEF
Shoulder and arm function	0.488, *p* = 0.001**	−0.670, *p* < 0.001**	+0.644, *p* < 0.001**
Mobility and ambulation	0.634, *p* < 0.001**	−0.680, *p* < 0.001**	+0.723, *p* < 0.001**
Fatigue	0.483, p = 0.001**	−0.570, *p* < 0.001**	+0.597, *p* < 0.001**
Cognitive function	0.240, *p* = 0.384, n.s	0.017, *p* = 0.915, n.s.	0.038, *p* = 0.814, n.s.
Activity limitation	0.605, *p* < 0.001**	−0.667, *p* < 0.001**	+0.668, *p* < 0.001**
Core strength and function	0.519, *p* = 0.001**	−0.656, *p* < 0.001**	+0.652, *p* < 0.001**
Eating function	0.438, *p* = 0.004**	−0.499, *p* = 0.001**	+0.355, *p* = 0.023*
Social performance	0.614, *p* < 0.001**	−0.682, *p* < 0.001**	+0.703, *p* < 0.001**
Body image	0.303, *p* = 0.055, n.s.	−0.52, *p* < 0.001**	+0.476, *p* = 0.002**
Hand and finger function	0.317, *p* = 0.044*	−0.548, *p* < 0.001**	+0.431, *p* = 0.003**
Social satisfaction	0.359, *p* = 0.021*	−0.515, *p* = 0.001**	+0.586, *p* < 0.001**
Pain	0.343, *p* = 0.028*	−0.398, *p* = 0.010*	+0.427, *p* = 0.005**
Emotional health	0.227, *p* = 0.154, n.s	−0.228, *p* = 0.152, n.s.	0.295, *p* = 0.062, n.s.
Communication	0.240, *p* = 0.131, n.s	−0.545, *p* < 0.001**	+0.399, *p* = 0.010**
Total FSHD-HI score	0.564, *p* < 0.001**	−0.708, *p* < 0.001**	+0.716, *p* < 0.001**
Short form	0.470, *p* = 0.002**	−0.634, *p* < 0.001**	+0.673, *p* < 0.001**

Correlations are shown as Spearman’s rho, *p*-value. *p < 0.05, **p < 0.01.

Criterion-related validity of FHSD-HI was decribed as correlation of FSHD-HI scores with SF-36 scores. In Table 5, we present only the strongest correlation between each SF-36 domain and the FSHD-HI domain, as well as correlation between each SF-36 domain and full FSHD-HI score. Intercorrelations between all domains are shown in Supplementary Table 3. In general, FSHD-HI total score correlated significantly with the total SF-36 score (rho = −0.733, *p* < 0.01), PCS (rho = −0.738, *p* < 0.01) and MCS (rho = −0.653, *p* < 0.01).

**Table 5:** Criterion-related validity (correlation between FSHD-HI subscores with SF-36 subscores).

SF-36 score	Mean ± SD	Correlation with FSHD-HI scores (rho)
PF	62.6 ± 31.2	Mobility and ambulation (−0.795, *p* < 0.001**)
		Total (−0.698, *p* < 0.001**)
RP	51.9 ± 44.6	Activity limitation (−0.709, *p* < 0.001**)
		Total (−0.667, *p* < 0.001**)
BP	62.4 ± 30.6	Pain (−0.692, *p* < 0.001**)
		Total (−0.426, *p* = 0.007**)
GH	51.6 ± 25.0	Fatigue (−0.720, *p* < 0.001**)
		Total (−0.688, *p* < 0.001**)
VT	57.3 ± 21.2	Emotional health (−0.760, *p* < 0.001**)
		Total (−0.667, *p* < 0.001**)
SF	74.3 ± 24.8	Cognitive function (−0.477, *p* < 0.001**)
		Total (−0.365, *p* = 0.022*)
RE	60.7 ± 44.5	Fatigue (−0.632, *p* < 0.001**)
		Total (−0.592, *p* < 0.001**)
MH	70.5 ± 21.6	Emotional health (−0.615, *p* < 0.001**)
		Total (−0.329, *p* = 0.041*)

PF, Physical Function; RP, Role Physical, BP, Body Pain; GH, General Health; VT, Vitality; SF, Social Functioning; RE, Role Emotional; MH, Mental Health. Correlations are shown as Spearman’s rho, *p*-value. *p < 0.05; **p < 0.01; only FSHD-HI domains with significant correlations are listed.

## Discussion

Our data demonstrated that the validated Serbian version of the FSHD Health Index (FSHD-HI-RS) is an understandable, easy-to-administer, and reliable patient-reported outcome measure (PROM). The translation and cultural adaptation required only minor sentence reformulations, indicating a high degree of conceptual equivalence with the original English version and suggesting that the challenges faced by Serbian patients in the areas of physical, social and disease specific health are similar to those observed in US cohort. During creation of FSHD-HI, the most prevalent and impactful symptoms in FSHD were detected through national US cross sectional study. Problems associated with mobility, activity and social role were most frequently mentioned by US participants (7).

The Serbian FSHD-HI showed excellent reliability, with an intraclass correlation coefficient (ICC) of 0.91, aligning well with findings from other validation studies of the original FSHD-HI, which report similarly high ICCs (e.g., >0.90) (8, 12). This supports the robustness and reproducibility of the questionnaire across languages and cultural contexts.

The median total FSHD-HI score in our cohort was 17.9, indicating a mild-to-moderate disease burden in our sample population. This value is lower, i.e., better, in comparison to Italian and Japanese studies that reported median or mean scores in higher range (4, 8). Those variations may occur due to sample characteristics, healthcare access, or cultural perceptions of symptoms. Differences might be explained by variability in disease progression, diagnostic timing, or study group selection.

Importantly, the wide interquartile range of the total FSHD-HI score (IQR: 2.15–51.95) seen in our cohort (Table 2) highlights the questionnaire’s ability to capture the full spectrum of the disease severity. While IQRs from other countries tend to be somewhat narrower (e.g., 10–45) (4, 8), the Serbian version’s wider range suggests that the questionnaire is capturing both mild and severely affected patients, which can be particularly useful in clinical and research settings for monitoring disease progression or stratifying patients in clinical trials.

The worst FSHD-HI scores in our cohort were observed with the subscales that measure shoulder and arm function, mobility and ambulation, and fatigue (Table 2). This is in line with the fact that FSHD is primarily a muscle disease that has a marked effect on these symptomatic domains. In our population, participants were least affected by limitations related to their cognition and eating function (as measured by these FSHD-HI subscales). Varma A et al. reported similar findings in USA cohort, as did researchers evaluating Italian and Japanese cohorts using the FSHD-HI (4, 8, 12). Although these domains generate less disease burden compared to other domains measured by the FSHD-HI, they are non-the-less important and occur in many FSHD patients (10). Indeed, the FSHD-HI can capture disease-specific features that are often overlooked or omitted by using generic QoL questionnaires, including those for neuromuscular diseases. We confirm data from previous validation studies that have shown that FSHD-HI is capable of simultaneously measuring disease burden involving many symptomatic areas as well as extraskeletal manifestations of FSHD (12).

Ceiling effect was not observed for FSHD-HI-RS suggesting that further worsening of FSHD during the disease course can be captured. This is in line with Japanese and Italian validation studies that reported low ceiling effect as well (4, 8), which indicates that the FSHD-HI can effectively capture the full spectrum of FSHD severity without losing sensitivity at the upper end of the scale. This highlights one of the strengths of the FSHD-HI for use in prospective natural history studies and therapeutic studies. Interestingly, we found that there was a slightly higher floor effect in the short form score compared to the full scale score. This characteristic may favor the use of the full instrument in future trials where patients are allotted adequate amounts of time to complete a key outcome.

We found that the FSHD-HI was able to differentiate between those with disease duration (Table 4), i.e., worse FHSD-HI scores was related to longer disease duration. This is in line with the fact that FSHD is a progressive disorder and that earlier onset of disease is generally associated with a more severe phenotype (18). We found a correlation of FHSD-HI-RS total score with MRC-SS and CCEF score (Table 4), confirming the fact that FSHD-HI is a patient-reported outcome measure that can capture and quantify differences in objective disease status. In other words, FSHD-HI-RS is capable of correctly measuring disease burden in a spectrum of FSHD patients from very mild to seriously functionally impaired. Other studies similarly have consistently demonstrated strong correlations between the FSHD-HI and the MRC-SS (4, 8), supporting its validity as a patient-reported outcome that aligns well with objective clinical measures of disease severity. To our knowledge, no previous studies did a corelation between CCEF total clinical score and FSHD-HI total score. Thus, our results using CCEF confirmed an association of the disease severity and FSHD-HI.

FSHD-HI total score also significantly correlated with SF-36 total score, PCS and MCS (Table 5) suggesting that FSHD-HI has the capability to capture both physical and emotional domains of quality of life in FSHD. The Italian validation study showed similar results regarding a good correlation between the total FSHD-HI-IT scale and SF-36 general health and physical activities (8). Similarly, a Japanese validation study investigated a correlation of FSHD-HI-J with the SF-36, World Health Organization Quality of Life Assessment, and QoL measure designed for neuromuscular diseases (INQoL). Their results showed significant correlation between FSHD-HI-J and above mentioned questionnaires (4).

There are several limitations of this study. First, the sample size was relatively small, which may limit the statistical power. However, FSHD is a rare disease with estimated prevalence of around 4 in 100,000 people (19). Since UCCS covers population of approximately 2.5 million as a tertiary center, we expect to have 100 FSHD cases in this population. Thus, our 41 cases represents almost half of all expected patients making our cohort representative. The patients included in this study were well motivated to participate and had the resources to attend a visit to our outpatient clinic, causing potential selection bias. It might be possible that the results from the patients who do not attend regular follow-up visits would be different from those we observed. Finally, longitudinal FSHD-HI-RS data and their relation to the disease progression are needed.

## Conclusion

Our data demonstrated that the validated Serbian version of FSHD-HI (FSHD-HI-RS) is an understandable, reliable, and valid QoL measure for use in FSHD patients. It is easy to administer and can capture disease-specific features, including extraskeletal manifestations of FSHD, that can be easily overlooked or omitted by using generic and even standard QoL questionnaires specific for neuromuscular diseases. Future research is needed to provide data about its efficacy in measuring disease progression over time and therapeutic response.

## Data Availability

The original contributions presented in the study are included in the article/Supplementary material, further inquiries can be directed to the corresponding author.
