# Supplementary material for: Disease burden in Serbian patients with facioscapulohumeral muscular dystrophy
Source: Front Neurol. 2026 Mar 16;17:1720488. doi: 10.3389/fneur.2026.1720488 (PMC13033487; doi:10.3389/fneur.2026.1720488)
Supplement: Supplementary file 1 [file Supplementary_file_1.docx]

**SUPPLEMENTS**

**Supplementary Table 1. Association of FSHD-HI with gender, age and education (construct related validity)**

| **FSHD HI domain** | **Gender**  **males vs females (mean ± SD), p** | **Age**  Spearman’s rho, p | **Education**  Spearman’s rho, p |
| --- | --- | --- | --- |
| Shoulder and arm function | 45.72 ± 35.1 vs 27.16 ± 34.5, p=0.101 | 0.018, p=0.909 | -0.107, p=0.504 |
| Mobility and ambulation | 39.89 ± 38.7 vs 26.40 ± 37.4, p=0.271 | 0.291, p=0.065 | -0.159, p=0.322 |
| Fatigue | 39.01 ± 34.0 vs 28.50 ± 34.3, p=0.338 | 0.146, p=0.362 | -0.144, p=0.370 |
| Cognitive function | 10.7 ± 22.6 vs 3.87 ± 7.3, p=0.238 | 0.134, p=0.403 | 0.011, p=0.948 |
| Activity limitation | 37.05 ± 35.5 vs 19.94 ± 34.0, p=0.130 | 0.221, p=0.164 | -0.102, p=0.525 |
| Core strength and function | 33.69 ± 32.96 vs 24.3 ± 35.16, p=0.388 | 0.074, p=0.645 | -0.174, p=0.278 |
| Eating function | 6.23 ± 15.99 vs 6.05 ± 14.3, p=0.970 | 0.093, p=0.564 | -0.225, p=0.157 |
| Social performance | 31.75 ± 29.02 vs 20.09 ± 30.6, p=0.223 | 0.145, p=0.365 | -0.157, p=0.328 |
| Body image | 34.79 ± 31.97 vs 21.01 ± 26.6, p=0.154 | -1.77, p=0.269 | -0.234, p=0.141 |
| Hand and finger function | 16.94 ± 28.78 vs 12.59 ± 20.94, p=0.559 | 0.111, p=0.489 | -0.182, p=0.255 |
| Social satisfaction | 25.07 ± 28.94 vs 15.26 ± 22.97, p=0.253 | 0.034, p=0.832 | -0.250, p=0.115 |
| Pain | 18.28 ± 22.8 vs 28.47 ± 36.1, p=0.274 | 0.162, p=0.311 | -0.105, p=0.514 |
| Emotional health | 19.52 ± 24.3 vs 12.88 ± 16.4, p=0.333 | -0.26, p=0.872 | -0.217, p=0.174 |
| Communication | 11.66 ± 17.4 vs 8.52 ± 14.66, p=0.548 | -0.97, p=0.546 | -0.210, p=0.187 |
| Total FSHD-HI score | 31.39 ± 26.1 vs 21.22 ± 27.1, p=0.233 | 0.080, p=0.621 | -0.171, p=0.284 |
| Short form | 32.83 ± 25.9 vs 22.16 ± 26.4, p=0.204 | 0.111, p=0.490 | -0.233, p=0.142 |

**Supplementary Table 2. Correlation of D4Z4 repeat length with FSHD-HI-RS scores (criterion-related validity)**

| **FSHD HI domain** | **D4Z4 repeat length** |
| --- | --- |
| Shoulder and arm function | -0.67, p=0.682 |
| Mobility and ambulation | -0.183, p=0.259 |
| Fatigue | -0.101, p=0.536 |
| Cognitive function | -0.09, p=0.954 |
| Activity limitation | -0.086, p=0.597 |
| Core strength and function | -0.063, p=0.698 |
| Eating function | 0.073, p=0.654 |
| Social performance | -0.055, p=0.737 |
| Body image | -0.035, p=0.832 |
| Hand and finger function | 0.065, p=0.691 |
| Social satisfaction | -0.017, p=0.917 |
| Pain | 0.033, p=0.841 |
| Emotional health | 0.038, p=0.817 |
| Communication | -0.057, p=0.726 |
| Total FSHD-HI score | -0.079, p=0.630 |
| Short form | -0.049, p=0.764 |

Correlations are shown as Spearman’s rho, p value.

**Supplementary Table 3.**  **Correlations between FSHD-HI-RS scores and SF-36 sscores (concurrent validity)**

| **SF-36 subscores** | **Correlation with FSHD-HI scores (rho)** |
| --- | --- |
| PF | Mobility and ambulation (-0.795, p<0.001**)  Short form (-0.715, p<0.001**)  Shoulder and Arm Function (-0.581, p<0.001**)  Fatigue (-0.686, p<0.001**)  Cognitive Function (-0.222, p=0.174)  Activity Limitation (-0.686, p<0.001**)  Core Strength and Function (-0.641, p<0.001**)  Gastrointestinal Function (-0.267, p=0.101)  Social Performance (-0.748, p<0.001**)  Body Image (-0.473, p=0.002**x)  Hand and Finger Function (-0.475, p=0.002*)  Social Satisfaction (-0.562, p<0.001**)  Pain (-0.545, p<0.001**)  Emotional Health (-0.350, p=0.029*)  Communication (-0.285, p=0.078)  Total (-0.698, p<0.001**) |
| RP | Activity limitation (-0.709, p<0.001**)  Short form (-0.715, p<0.001**)  Shoulder and Arm Function (-0.591, p<0.001**)  Mobility and Ambulation (-0.602, p<0.001**)  Fatigue (-0.695, p<0.001**)  Cognitive Function (-0.365, p=0.022*)  Core Strength and Function (-0.564, p<0.001**)  Gastrointestinal Function (-0.181 p=0.271)  Social Performance (-0.578, p<0.001**)  Body Image (-0.397, p=0.012*)  Hand and Finger Function (-0.544, p<0.001** )  Social Satisfaction (-0.458, p<0.003*)  Pain (-0.592, p<0.001**)  Emotional Health (-0.381, p=0.17)  Communication (-0.258, p=0.113)  Total (-0.667, p<0.001**) |
| BP | Pain (-0.692, p<0.001**)  Short form (-0.461, p=0.03*)  Shoulder and Arm Function (-0.242, p=0.138)  Mobility and Ambulation (-0.427, p=0.07)  Fatigue (-0.521, p<0.001**)  Cognitive Function (-0.233, p=0.154)  Activity Limitation (-0.365, p=0.022*)  Core Strength and Function (-0.323, p=0.045*)  Gastrointestinal Function (-0.130 p=0.431)  Social Performance (-0.435, p=0.006*)  Body Image (-0.160, p=0.330)  Hand and Finger Function (-0.506, p<0.001**)  Social Satisfaction (-0.365, p=0.022*)  Emotional Health (-0.329, p=0.041*)  Communication (-0.228, p=0.164)  Total (-0.426, p=0.007**) |
| GH | Fatigue (-0.720, p<0.001**)  Short form (-0.728, p<0.001**)  Shoulder and Arm Function (-0.597, p<0.001**)  Mobility and Ambulation (-0.607, p<0.001**)  Cognitive Function (-0.324, p=0.044)  Activity Limitation (-0.708, p<0.001**)  Core Strength and Function (-0.551, p<0.001**)  Gastrointestinal Function (-0.263, p=0.106)  Social Performance (-0.620, p<0.001**)  Body Image (-0.527, p<0.001**)  Hand and Finger Function (-0.549, p<0.001**)  Social Satisfaction (-0.648, p<0.001**)  Pain (-0.516, p<0.001**)  Emotional Health (-0.571, p<0.001**)  Communication (-0.264, p=0.104)  Total (-0.688, p<0.001**) |
| VT | Emotional health (-0.760, p<0.001**)  Short form (-0.633, p<0.001**)  Shoulder and Arm Function (-0.536, p<0.001**)  Mobility and Ambulation (-0.573, p<0.001**)  Fatigue (-0.715, p<0.001**)  Cognitive Function (-0.428, p=0.007*)  Activity Limitation (-0.612, p<0.001**)  Core Strength and Function (-0.615, p<0.001**)  Gastrointestinal Function (-0.130, p=0.429)  Social Performance (-0.661, p<0.001**)  Body Image (-0.614, p<0.001**)  Hand and Finger Function (-0.594, p<0.001**)  Social Satisfaction (-0.672, p<0.001**)  Pain (-0.589, p <0.001**)  Communication (-0.204, p=0.214)  Total FSHD-HI Score (-0.667, p<0.001**)  Total (-0.667, p<0.001**) |
| SF | Cognitive function (-0.477, p<0.001**)  Short form (-0.445, p=0.005*)  Shoulder and Arm Function (-0.240, p=0.141)  Mobility and Ambulation (-0.274, p=0.092)  Fatigue (-0.467, p=0.003*)  Activity Limitation (-0.289, p=0.075)  Core Strength and Function (-0.288, p=0.075)  Gastrointestinal Function (0.062, p=0.707)  Social Performance (-0.348, p =0.030*)  Body Image (-0.385, p=0.016*)  Hand and Finger Function (-0.405, p=0.010*)  Social Satisfaction (-0.408, p=0.010*)  Pain (-0.466, p=0.003*)  Emotional Health (-0.520, p<0.001**)  Communication (-0.157, p=0.339)  Total (-0.365, p=0.022*) |
| RE | Fatigue (-0.632, p<0.001**)  Short form (-0.628, p<0.001**)  Shoulder and Arm Function (-0.524, p<0.001**)  Mobility and Ambulation (-0.521, p<0.001**)  Cognitive Function (-0.461, p=0.003*)  Activity Limitation (-0.556, p<0.001**)  Core Strength and Function (-0.539, p<0.001**)  Gastrointestinal Function (-0.200, p=0.222)  Social Performance (-0.499, p<0.001**)  Body Image (-0.454, p=0.004*)  Hand and Finger Function (-0.526, p<0.001**)  Social Satisfaction (-0.481, p=0.002)  Pain (-0.524, p<0.001**)  Emotional Health (-0.542, p<0.001**)  Communication (-0.308, p=0.57)  Total FSHD-HI Score (0.592, p<0.001**) |
| MH | Emotional health (-0.615, p<0.001**)  Short form (-0.321, p=0.046*)  Shoulder and Arm Function (-0.250, p=0.124)  Mobility and Ambulation (-0.149, p=0.366)  Fatigue (-0.398, p=0.012*)  Cognitive Function (-0.390, p=0.014*)  Activity Limitation (-0.274, p=0.092)  Core Strength and Function (-0.234, p=0.151)  Gastrointestinal Function (-0.012, p=0.942)  Social Performance (-0.267, p=0.100)  Body Image (-0.419, p=0.01*)  Hand and Finger Function (-0.369, p=0.021*)  Social Satisfaction (-0.487, p= 0.02*)  Pain (-0.342, p=0.033*)  Communication (-0.124, p=0.453)  Total (-0.329, p=0.041*) |

PF – Physical Function, RP – Role Physical, BP – Body Pain, GH – General Health, VT - Vitality, SF – Social Functioning, RE – Role Emotional, MH – Mental Health. Correlations are shown as Spearman’s rho, p value. * p<0.05; ** p<0.001
